# Quality of medical service, patient satisfaction and loyalty with a focus on interpersonal-based medical service encounters and treatment effectiveness: a cross-sectional multicenter study of complementary and alternative medicine (CAM) hospitals

**DOI:** 10.1186/s12906-017-1691-6

**Published:** 2017-03-28

**Authors:** Chang Eun Kim, Joon-Shik Shin, Jinho Lee, Yoon Jae Lee, Me-riong Kim, Areum Choi, Ki Byung Park, Ho-Joo Lee, In-Hyuk Ha

**Affiliations:** Jaseng Spine and Joint Research Institute, Jaseng Medical Foundation, 858 Eonju-ro, Gangnam-gu, Seoul, Republic of Korea

**Keywords:** Health care facilities, manpower, and services, Health services, Health personnel, Treatment outcome, Health care quality, access, and evaluation, Patient satisfaction, Surveys and questionnaires

## Abstract

**Background:**

Treatment effectiveness holds considerable importance in the association between service quality and satisfaction in medical service studies. While complementary and alternative medicine (CAM) use grows more prominent, comprehensive evaluations of the quality of medical service at CAM-oriented hospitals are scarce. This study assesses the quality of medical services provided at a CAM-oriented hospital of Korean medicine using the service encounter system approach and analyzes the influence of treatment effectiveness on patient loyalty.

**Methods:**

A survey study using one-on-one interviews was conducted using a cross-sectional design in outpatients visiting one of fifteen Korean medicine facilities located throughout Korea. A total of 880 surveys were completed from June to July, 2014, and 728 surveys were included in the final analysis after excluding incomplete or incorrect questionnaires. The reliability and validity of the surveys was confirmed using Cronbach’s alpha coefficient and confirmatory factor analysis, and a structural equation modeling analysis was performed to verify causality and association between factors (quality of medical service, treatment effectiveness, patient satisfaction, and intent to revisit).

**Results:**

The measured factors of physician performance and quality of service procedures had a positive effect on treatment effectiveness. The impression of the facilities and environment directly impacted satisfaction rates for interpersonal-based medical service encounters, while treatment effectiveness positively affected satisfaction regarding quality of medical service. However, treatment effectiveness had a more significant effect on satisfaction compared to facilities and environment, and it indirectly affected satisfaction and directly influenced intent to revisit. Treatment effectiveness and satisfaction both positively influenced intent to revisit.

**Conclusions:**

The importance of treatment effectiveness should be recognized when examining quality of medical services, and we hope that these findings may contribute to future studies.

**Electronic supplementary material:**

The online version of this article (doi:10.1186/s12906-017-1691-6) contains supplementary material, which is available to authorized users.

## Background

The contemporary concept of service quality refers to the comparison of perceived expectations with perceived performance of a specific service, and may therefore be considered to be the difference between perceived expectations and performance [1]. This conceptualization of service quality has its roots in the expectancy-disconfirmation paradigm [2]. In recent years, patient interest in the quality of medical services has risen gradually in medical service encounters. While securing access to medical services was the main focus of patient needs in the past, patients are now exposed to more options due to increase in supply, and in response to this hospitals are increasing their efforts to improve quality of medical services. The concept of patient satisfaction has been introduced to the medical community from increased interest in medical service quality improvement and is part of an effort to refine medical services [3]. Similar to how companies produce products and render services in response to consumer demand, hospitals may be likened to companies and their patients to customers, offering medical services as goods. From this viewpoint, hospitals should aim to provide optimal medical care and services customized to individual patients to draw more return visits. In light of this heightened interest in medical service quality, numerous studies examining the association between hospital service quality and patient satisfaction have been published [4, 5].

International interest in complementary and alternative medicine (CAM) is growing with many patients reporting use of both conventional and CAM treatment [6]. Following an increase in the number of Korean medicine hospitals, Korean medicine hospitals are devoting more time and effort into quality improvement of medical services. However, the number of related studies does not reflect this pattern and the studies that are currently available are mostly of low quality. A 2010 systematic review on Korean medicine service usage and satisfaction of Korean medicine facilities in Korea [7] assessed 17 studies published between 1991 and 2007. Most studies were conducted in small samples and were geographically concentrated which limited generalizability. In addition, most of the data was analyzed by t-test or Chi-square test which confined analyses to intergroup differences and was not suitable for determining the causal factors underlying between-group differences. Validity and reliability were also difficult to verify because many studies chose to forgo factor analysis of questionnaire items and assessment of multicollinearity among independent factors [7]. There is a dearth of studies reporting on the service quality of CAM-oriented medical facilities, and most studies only examine the quality of medical services in conventional medicine users with very few addressing Korean medicine, implying that Korean medicine facilities require a more systematic approach to service quality improvement [8].

### Evaluation of service quality focusing on the service encounter system

The interaction between service provider and consumer plays a significant role in valuation of the quality of services provided. Familiarity and encounter opportunities between service provider and consumer also factor strongly in customer evaluations. This is due to the inseparability of production and consumption in services, and the fact that consumers tend to perceive service encounters as part of the provided services along with other tangible factors such as facilities [9].

One of the reasons why the service encounter system deserves more emphasis in service quality assessment is that though SERVQUAL, the most widely used service quality evaluation tool, grades service quality based on 5 values (reliability, responsiveness, assurance, empathy, and tangibles) [10], which parties are responsible for outcomes and subsequent revision may be unclear, thus proving service improvement difficult.

A recent study by Chang et al. explains how the concept of service quality has evolved into an interpersonal relationship-based medical service and accordingly evaluated service quality and satisfaction with focus on the service encounter by professional personnel, general administrative personnel, and environment and space [5]. The items of the current survey study are also constructed focusing on service encounters with service providers. The term medical service encompasses both medical treatment offered by the healthcare provider (comprising basic services of the medical institution) and supplementary services (entailing concomitant services relating to patient care). In terms of individual medical services, these factors should correlate to services proffered by physicians, nurses, administrative personnel, and facilities and environment, and service procedures.

### Service quality and patient satisfaction focusing on treatment effect

Most of the studies on medical service quality and satisfaction are concerned with overall services and do not consider the link between medical service-specific variables such as patient satisfaction and treatment effectiveness. As the primary objective of patients visiting healthcare facilities is to receive treatment, we hypothesized that regardless of excellent service quality, if the treatment effectiveness is poor, satisfaction and loyalty would be negatively affected.

Treatment results impact the relationship between patient and hospital and may result in a patient response that ranges from satisfactory to taking legal action for medical malpractice depending on treatment results [11]. Therefore, increase in treatment effectiveness may bring about closer treatment-based relationships between hospital and patient, potentially leading to higher satisfaction.

On a different note, the quality of medical service may also affect the interpretation of treatment effect as actual treatment effects not only include tangible treatment outcomes but also nonspecific psychological aspects such as placebo effect and rapport. Treatment expectations are known to greatly influence placebo effect, and because of this the expectations of interventions are frequently measured and accounted for in clinical trials [12]. Recently, several reports on underlying mechanisms of expectation using such tools as brain imaging have been published [13]. It can be reasonably inferred that expectations and other psychological factors may highly impact the quality of services in service encounters.

Previous literature on marketing shows that about half of satisfied customers repurchase goods, suggesting the need for closer management of loyalty indices [14]. Customer loyalty is defined as the intent to repeatedly use a certain company or store [15], which would be equivalent to revisiting a certain hospital with regard to patient loyalty [16]. Loyalty is generally assessed based on the willingness to use a product again, and in the medical service sector, the intent to revisit a hospital or to recommend the facilities to others is measured [17].

Based on the above, the following hypotheses were suggested:

Hypothesis 1: Service quality may positively influence treatment effectiveness.

Hypothesis 2: Service quality may positively influence satisfaction.

Hypothesis 3: Treatment effect may positively influence satisfaction.

Hypothesis 4: Treatment effect may positively influence revisit behavior.

Hypothesis 5: Satisfaction may positively influence revisit behavior.

### Structural model based on theoretical relationship

**Fig. 1:**
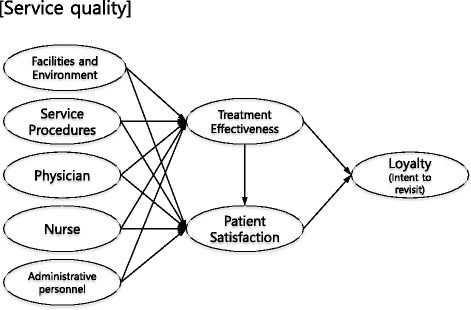
Initial structural model drawn based on theoretical relationships. Service quality = facilities and environment, service procedures, physician, nurse, and administrative personnel

We hypothesized that various factors of medical service would reflect positively on treatment effect and patient satisfaction, and that treatment effectiveness and satisfaction would positively influence the intent to revisit given previous findings. The structural model drawn from this hypothetical base is given in Fig. 1.

## Methods

### Research design and participants

This survey study was carried out at 15 locations of Jaseng Hospital of Korean medicine, a Korean medicine hospital specializing in spine care. The sites included 3 hospitals designated by the Korean Ministry of Health and Welfare to specialize in spinal disorders in 2014 (2nd accreditation term of specialty Korean medicine hospitals), and the first and only Korean medicine hospital to be recognized to specialize in spinal conditions in the 1st term from 2011. Specialty hospitals are hospitals certified by the Korean Ministry of Health and Welfare to provide highly-skilled, advanced medical treatment for specific specialties/disorders (Korean medical law Act 3, Clause 5). Jaseng Hospital is comprised of 19 hospital sites and clinics in Korea and 6 clinics in the United States as of 2017, treating over 900,000 cases per year with integrative Korean medicine treatment (acupuncture, Chuna manual therapy, herbal medicine, and pharmacopuncture). Previous studies give further details regarding the integrative care provided at this institution and have demonstrated favorable outcomes for various spine conditions [18–20].

According to a study by Hair et al. on sampling multivariate data, the sample size should be at least 5–10 times the number of study variables and each variable sampling should be ≥30 [21]. We therefore set the sample size at 50 if the hospital/clinic had <100 inpatient beds and at 100 if it had ≥100 beds. Thirty samples were collected from the Pyeongchon clinic, the smallest of the branches.

Three trained surveyors were selected to survey 4 districts (Seoul, Gyeonggi, Chungcheong, and Gyeongsang) using one-on-one interviews at each site. Though one-on-one interviews increases the need for higher surveyor education and managing costs, greater time commitment, and more interrater variability, they effectively prevent loss of data. One-on-one interviews also allow the surveyor to assist with survey comprehension, resulting in higher reliability and increasing accessibility to young and elderly surveyees, surveyees with reading (including illiteracy) or hearing disabilities, and those with lower education. For these reasons, one-on-one interviews were used to more accurately capture patient satisfaction regarding medical service use. In addition, surveyors received training to standardize survey processes and methods, minimize error, and ensure that the same meaning was conveyed for each item to reduce false or missing values. A designated administrator regularly checked survey progress for management purposes.

Outpatient samples were selected by convenience sampling. A total 880 questionnaires were completed from June to July 2014. After incomplete and incorrect questionnaires were excluded, 728 were included for final analysis. To protect personal information and encourage expression of free opinion, names, addresses, and diagnostic data of the surveyees were not collected.

### Instrument

The method of questionnaire modification and development is based on the process of using questionnaire dimensions initially obtained from pre-existing questionnaires and then further developing the questionnaires to better accommodate research objectives and industry characteristics [22–24].

Medical service quality was conceptualized through the evaluation of perceived performance in the following five areas of patient services: facilities and environment, service procedures, and physician, nurse, and administrative personnel performance. The final instrument consists of six items covering facilities and environment, eight items on service procedure, six items on physician performance, five items on nursing staff performance, and five items on administrative personnel. The performance section constituted of thirty items in total. Further, four items addressed treatment effect, two items were included on customer satisfaction, and lastly two items examined the patient’s revisit intent, covering a total of eight construct areas (total 38 items). Each item was rated on 5-point Likert scales ranging from 1, indicating “very dissatisfied” to 5, “very satisfied”.

The questionnaire used in this study was originally based on SERVQUAL. It was modified to concentrate on interpersonal relationship-based medical service evaluation in the service encounter system. The modification reflected expert opinion that the quality of Korean medicine service would be more affected by medical service providers (e.g. physicians, nursing staff) than conventional medicine as it is less influenced by technological advances or facilities. Moreover, satisfaction with service quality does not always correlate with the treatment effectiveness of a medical service. In addition to the theory that better service quality would lead to improved satisfaction and subsequent loyalty, we hypothesized that treatment effectiveness would affect both patient loyalty and service quality satisfaction in medical service, and accordingly added items on treatment effectiveness. In summary, we modified the basic SERVQUAL format with focus on the following:

1. Interpersonal relationship-based medical service (physician, nursing staff, and administrative personnel)
2. Effect of service quality on treatment effectiveness
3. Assessment of the effect of service quality satisfaction and treatment outcome on intent to revisit, respectively

The full questionnaire contents are available in Additional file 1.

### Data analysis

Analyses were conducted using SPSS 21.0 and AMOS 21.0 statistical software packages (IBM Corporation, Armonk, NY, USA), and basic participant characteristics are presented as descriptive statistics.

Prior to validating the hypotheses with structural equation modeling (SEM), the reliability and validity of the measurement instrument (consumer satisfaction questionnaire) was verified. Reliability was evaluated with Cronbach’s alpha (α) coefficient where a value of ≥0.7 is generally considered to be reliable [21]. Validity was verified through confirmatory factor analysis (CFA). Convergent validity is considered good if the standardized factor loading is >0.5, average variance extracted (AVE) is >0.5, and construct reliability is >0.7. Discriminant validity is confirmed if √AVE > latent variable correlation coefficient (ϕ) or the result of ϕ^2^ ± 2 × standard error (SE) does not include 1 [25]. Nomological validity is verified by assessing whether correlations among variables are positively or negatively significant.

Structural equation modeling was performed using maximum-likelihood-estimation to test the significance of the causal relationship among medical service quality factors and treatment effectiveness, customer satisfaction, and loyalty (intent to revisit). Chi-square test (*χ* ^2^), RMR, RMSEA, GFI, NFI, IFI, TLI, CFI, and AIC were used to test the model’s goodness of fit [26]. Path coefficients of exogenous latent variables which influence endogenous latent variables were additionally compared, and significance testing was performed on indirect effect of latent variables using bootstrapping method.

### Ethical approval

Surveys were conducted without participant signatures nor other identifiers linking data to personal information. The purpose of the survey and privacy protection policy was stated at the beginning of the questionnaire, and filling out the survey was considered to constitute informed consent. Study data were used in accordance with the Declaration of Helsinki revised in 2013. The study was approved by the Institutional Review Board (IRB) of Jaseng Hospital of Korean medicine (KNJSIRB2016–003).

## Results

### Subject characteristics

Study participants were recruited from outpatient care, of which 60.7% were female, and 38.2% male. In age groups, 13.6% were in their 20s, 24.7% were in their 30s, 24.0% were in their 40s, 19.2% were in their 50s, and 17.7% were in their 60s or older; showing that the 30s to 40s age group were predominant. Regarding occupation, approximately one-third (36.5%) were housewives, 27.7% were corporate workers, 10.0% were entrepreneurs, 8.1% were students, 4.4% were civil servants, and 9.8% were employed otherwise, with housewives taking up the largest percentage. In number of visits, the majority (78.6%) had visited ≥5 times, 2.6% were on their first visit, 6.7% were visiting a second time, 6.6% a third, and 5.4% for a fourth time (Table 1).

**Table 1:** Characteristics of survey participants (*n* = 728)

Variable	Frequency (*n*)	Percentage (%)
Sex		
Male	278	38.2
Female	442	60.7
Age		
≤29 years	99	13.6
30–39 years	180	24.7
40–49 years	175	24.0
50–59 years	140	19.2
≥60 years	129	17.7
Occupation		
Student	59	8.1
Housewife	266	36.5
Corporate worker	202	27.7
Civil servant	32	4.4
Entrepreneur	73	10.0
Other	71	9.8
Number of visit(s)		
First time	19	2.6
Second time	49	6.7
Third time	48	6.6
Fourth time	39	5.4
≥Fifth time	572	78.6

### Construct validity and reliability

Observed variable 1 (“convenience of the hospital location and transportation”) and 2 of the facilities and environment section (“convenience of the parking facility”), and observed variable 4 of the treatment effect section (“treatment cost is appropriate”) showed low standardized factor loading (0.38, 0.49, and 0.46, respectively), impairing convergent validity, and observed variable 2 of the facilities and environment section had to be excluded due to a high rate of missing values. Variable 2 of patient satisfaction (“the provided treatment service was satisfactory considering treatment cost”) was also excluded from analysis as Cronbach’s alpha (α) coefficient for patient satisfaction did not meet standards at 0.67 and the correlation coefficient with intent to revisit was very high at 0.86, resulting in nonsatisfaction of discriminant validity requirements and low standardized factor loading (Additional file 1).

Upon exclusion of irrelevant variables, all latent variables displayed Cronbach’s alpha (α) coefficients of 0.76 to 0.95, and all satisfied reliability [25, 27]. As convergent validity was above standard value, convergent validity was considered to be satisfactory (Table 2). The discriminant validity also met the statistical standard calculated from latent variables. We found positive correlations between all latent variables, which was as we had hypothesized, indicating that nomological validity was satisfied (Table 3).

**Table 2:** Validity and reliability of questionnaire constructs and items

Construct (dimension)/Question item	Standard factor loading	AVE	CR	Cronbach’s alpha
Facilities and Environment		0.696	0.900	0.820
3. Hospital indoor temperature (air conditioning/heating) and ventilation was satisfactory.	0.806			
4. The hospital was clean and pleasant overall.	0.881			
5. The hospital was well-equipped with amenities (e.g. cafe, drink vending machine, water purifier, waiting space, cash machine).	0.678			
6. On-site hospital facilities were easy to locate (e.g. consultation room, diagnostic imaging department, physical therapy room, restroom).	0.606			
Service procedures		0.635	0.932	0.889
1. Making appointments was convenient.	0.707			
2. I was able to make appointments on the date and time I wanted.	0.705			
3. Staff was prompt in receiving and returning phone calls.	0.765			
4. The registration procedure for consultations was convenient.	0.820			
5. Adequate information on waiting time was given in advance.	0.683			
6. Waiting time duration of examination and treatment were acceptable.	0.597			
7. The payment process was convenient.	0.790			
8. Payment receipt items were easy to understand.	0.690			
Physician		0.887	0.979	0.946
1. The physicians were neat and tidy in appearance.	0.850			
2. The physicians were kind and courteous.	0.864			
3. Information on treatment was always given by physicians in advance.	0.864			
4. The physicians were attentive to my conversation (queries).	0.890			
5. The physicians gave sufficient explanation on symptoms and treatment plans that were easy to comprehend.	0.851			
6. The physicians commanded sufficient professional knowledge.	0.876			
Nursing staff		0.878	0.973	0.939
1. Nursing staff were neat and tidy in appearance.	0.893			
2. Nursing staff were kind and courteous.	0.901			
3. Nursing staff were attentive to my conversation (queries).	0.901			
4. Nursing staff gave sufficient explanation on symptoms and treatment plans that were easy to comprehend.	0.857			
5. Nursing staff commanded sufficient professional knowledge.	0.812			
Administrative personnel		0.901	0.978	0.954
1. Administrative personnel were neat and tidy in appearance.	0.845			
2. Administrative personnel were kind and courteous.	0.921			
3. Administrative personnel were attentive to my conversation (queries).	0.941			
4. Administrative personnel gave sufficient explanation on symptoms and treatment plans that were easy to comprehend.	0.913			
5. My queries (demands) were promptly taken care of.	0.877			
Treatment effectiveness		0.887	0.959	0.935
1. Treatment was effective.	0.898			
2. Treatment was reliable.	0.933			
3. Treatment and prescriptions were appropriate.	0.902			
Patient satisfaction				
1. I was satisfied with this hospital overall.				
Loyalty (Intent to revisit)		0.532	0.693	0.760
1. I intend to continue using this hospital.	0.710			
2. I would recommend this hospital to others.	0.864			

Service quality = facilities and environment, service procedures, physician, nurse, and administrative personnel

*CR* Construct reliability, *AVE* Average variance extracted, Satisfies CR > 0.7, AVE > 0.5

**Table 3:** Correlation between latent variables

Construct	1	2	3	4	5	6	7	8
1. Facilities and Environment	0.834^a^							
2. Service Procedures	0.723	0.797^a^						
3. Physician	0.585	0.640	0.942^a^					
4. Nurse	0.555	0.625	0.790	0.937^a^				
5. Administrative personnel	0.508	0.623	0.729	0.747	0.949^a^			
6. Treatment effectiveness	0.403	0.445	0.509	0.435	0.407	0.942^a^		
7. Satisfaction	0.431	0.444	0.469	0.429	0.384	0.622		
8. Loyalty (Intent to revisit)	0.407	0.452	0.459	0.393	0.365	0.701	0.680	0.730^a^

*p* < 0.05 is regarded to be significant in all correlation coefficients between factors

Service quality = facilities and environment, service procedures, physician, nurse, and administrative personnel

^a^value of diagonal line is AVE1/2

### Results of structural equation modeling (SEM)

The initial goodness of fit for the model constructed with observed variables selected through reliability and validity of the measurement instrument was *χ* ^2^ = 2972.4, RMR = 0.029, GFI = 0.786, AGFI =0.748, RMSEA =0.082, NFI = 0.782, IFI = 0.891, TLI = 0.879, CFI = 0.891, and AIC = 3152.4, and with the exception of RMR, did not meet suitability standards. We therefore chose to modify the model by maintaining variables while considering modification indices. First, starting from the largest modification index, error covariance among similar latent variables of theoretical relevance was set and added (service procedure 5 ↔ service procedure 6, service procedure 7 ↔ service procedure 8, physician 1 ↔ physician 2, nursing staff 4 ↔ nursing staff 5, administrative personnel 4 ↔ administrative personnel 5). Considering that the first item in the physician, nursing staff, and administrative personnel was the same, error covariance among different latent variables was allowed (physician 1 ↔ nurse 1, physician 1 ↔ administrative personnel 1, nurse 1 ↔ administrative personnel 1) and the model was modified accordingly. Goodness of fit of the final model was *χ* ^2^ = 1978.2, RMR = 0.03, GFI = 0.86, AGFI =0.83, RMSEA =0.06, NFI = 0.92, IFI = 0.94, TLI = 0.93, CFI = 0.93, and AIC = 2174.2. As *χ* ^2^ is greatly influenced by sample size and the current sample size is large, *χ* ^2^ is likely to have been overestimated. GFI and AGFI values are in close proximity to standard values and the other goodness of fit indices all meet appropriate standards, showing that AIC of the modified model is lower than that of the initial model and that the modified model is superior. It was therefore selected as the final model.

**Fig. 2:**
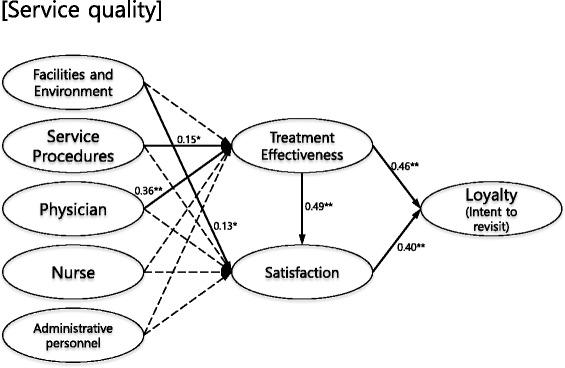
Final selected structural model with path coefficients. Service quality = facilities and environment, service procedures, physician, nurse, and administrative personnel

In the final model, the analysis results of the study hypotheses are presented as standardized path coefficients and significance levels. Factors significantly affecting treatment effectiveness were service procedure (β = 0.15) and physician performance (β = 0.36), and those significantly affecting patient satisfaction were facilities and environment (β = 0.13) and treatment effect (β = 0.49). Also, treatment effectiveness (β = 0.46) and patient satisfaction (β = 0.40) both significantly influenced intent to revisit (Table 4, Fig. 2). In influence of significant path coefficients, path comparison between the two models of physician and service procedure on treatment effectiveness was Δ*χ* ^2^ = 7.6, indicating that the physician factor had a statistically stronger influence on treatment effectiveness than service procedure. Path comparison between the two models of facilities and environment and treatment effectiveness on patient satisfaction was Δ*χ* ^2^ = 15.9, likewise implying that treatment effectiveness had a statistically stronger effect on patient satisfaction than facilities and environment. As the path comparison between the two models of treatment effectiveness and patient satisfaction on the intent to revisit was Δ*χ* ^2^ = 0.11, which is smaller than the standard value of 3.84, it can be inferred that treatment effectiveness and patient satisfaction have a similar impact on intent to revisit (Table 5).

**Table 4:** Path coefficient results in structural equation modeling

Path				Standard Coefficient	*t* value
H1	Facilities and Environment	(−->)	Treatment effectiveness	0.084	1.447
H2	Facilities and Environment	(−->)	Satisfaction	0.132*	2.631
H3	Service Procedures	(−->)	Treatment effectiveness	0.145*	2.29
H4	Service Procedures	(−->)	Satisfaction	0.031	0.563
H5	Physician	(−->)	Treatment effectiveness	0.358**	5.612
H6	Physician	(−->)	Satisfaction	0.077	1.37
H7	Nurse	(−->)	Treatment effectiveness	0.005	0.076
H8	Nurse	(−->)	Satisfaction	0.076	1.404
H9	Administrative personnel	(−->)	Treatment effectiveness	0.014	0.244
H10	Administrative personnel	(−->)	Satisfaction	−0.012	-0.25
H11	Treatment effectiveness	(−->)	Satisfaction	0.489**	13.476
H12	Treatment effectiveness	(−->)	Loyalty	0.456**	9.956
H13	Satisfaction	(−->)	Loyalty	0.397**	9.217

*χ* ^2^=1978.2, RMR = 0.03, GFI = 0.86, AGFI = 0.83, RMSEA = 0.06, NFI = 0.92, IFI = 0.94, TLI = 0.93, CFI = 0.93, AIC = 2174.2

Service quality = facilities and environment, service procedures, physician, nurse, and administrative personnel

*p < 0.05; **p < 0.001

**Table 5:** Comparison of path coefficients

			*χ* ^2^	df	∆*χ* ^2^
Unlimited Measurement Pattern			1978.18	497	-
Limited Measurement Pattern					
Service Procedure vs Physician	(−->)	Treatment effectiveness	1985.85	498	7.66*
Facilities and Environment vs Treatment effectiveness	(−->)	Satisfaction	1994.09	498	15.91*
Treatment effectiveness vs Satisfaction	(−->)	Loyalty	1978.29	498	0.11

*df* degree of freedom

*Statistically significant if the constraint model - unconstrained model (df= 1) ≥3.84

### Mediation effect analysis (direct, indirect, and total effect)

In addition, we assessed the direct, indirect, and total effects of service quality through mediating variables (treatment effect and satisfaction) on the final dependent variable, intent to revisit. Results showed that service procedure and physician performance had an indirect but significant impact on satisfaction with treatment effectiveness as the mediating variable, and facilities and environment, service procedure, physician performance, and treatment effectiveness had a significant but indirect effect on intent to revisit with satisfaction as the mediator. A noteworthy point is that though the physician factor did not have a direct effect on satisfaction, it commanded a significant effect indirectly through treatment effectiveness (Additional file 1).

## Discussion

This study investigated the validity of various medical service quality factors using the results from a customer satisfaction questionnaire and examined potential causal relationships among the measured indices of medical service quality, treatment effect, patient satisfaction, and intent to revisit. We also assessed which of the five medical service quality factors was most influential in treatment effectiveness and satisfaction.

Items relating to service procedure and physician performance were shown to positively affect treatment effectiveness through hypotheses testing, and physician-related factors had a stronger influence on treatment effectiveness than service procedure factors. Among medical service qualities, facilities and environment, and treatment effectiveness were shown to have positive effects on satisfaction, and upon testing the interaction hypothesis on treatment effectiveness, patient satisfaction, and intent to revisit, all hypothesized pathways from the study model were selected.

In conclusion, through analysis of overall causal relationships between medical service quality and treatment effectiveness, patient satisfaction, and intent to revisit, the pathways of medical service quality → treatment effect → patient satisfaction → intent to revisit, and medical service quality → patient satisfaction → intent to revisit were significant. Physician-related medical service quality factors appeared to have the strongest effect on treatment effectiveness and treatment effectiveness had the greatest impact on patient satisfaction.

Though a previous study by Zeithaml showed that perceived product quality influences product value [28] and Grewal et al. similarly illustrated how service quality positively affects service value [29], there is a distinct paucity of medical service studies assessing service quality with respect to treatment effectiveness. Many studies have been conducted on the importance of personal relationships and the role of service providers and customers in the service industry sector [30], and they generally report that strong personal bonds between the service provider and customer positively influence customer satisfaction and loyalty [31]. Within the medical service frame, the relationship between physician and patient could be seen to correspond with that of the service provider and customer.

Several previous studies agree that the performance of physicians and nurses are factors of highest importance in patient satisfaction [32–34], and Park reported that the physician is the most important factor to inpatients hospitalized at Korean medicine facilities, which is consistent with the findings of the present study [35]. Satisfaction is defined as the level of satisfaction or dissatisfaction with medical service after services have been provided, and denotes that patient satisfaction may act as a mediating variable between service quality and behavior intention [17]. These study results generally concur with previous study results that report on the effect of service quality on satisfaction [36]. In addition, a number of studies have commented on the importance of physical factors such as facilities and environment in service quality [37, 38].

More encounter opportunities are open to patients at Korean medicine hospitals due to the innate nature of Korean medicine compared to conventional medicine, with interventions such as acupuncture, moxibustion, cupping, and Chuna manual therapy all necessitating more time spent in patient interaction and allowing for stronger bonding between provider and patient. These aspects naturally affect building rapport with patients and influence treatment effectiveness.

Establishing a comfortable environment for treatment and equipping the hospital with service-friendly facilities is another important aspect for patient satisfaction [39]. Patients also need to feel that they can trust in their physicians and that their physicians are providing treatment based on sufficient knowledge. This trust enables the patient to feel comfortable and to achieve higher treatment success. In order for patients to feel that the total medical cost or time invested was worthwhile, a certain level of patient satisfaction has to be maintained from registration to treatment, and satisfaction commonly begets a stronger response to treatment. As this Korean medicine facility is a spine-specialty hospital, many patients presented with acute pain and compromised walking or movement, and prompt service and less time to treatment could potentially lead to faster resolution of pain in the patients’ perception.

Though the importance of service quality evaluation should be given higher recognition in medicine, there is some confusion as to which factors constitute medical service. Services mainly pertain to the human body and the amount and degree of involvement on the individual level is extensive [40]. Unlike prior studies covering the relatively straightforward causal pathway from service quality to satisfaction to loyalty, this study evaluated the overall relationship between service quality, treatment effectiveness, patient satisfaction, and intent to revisit. Previous medical service questionnaires developed from general service evaluation methods do not place sufficient weight on treatment effectiveness. In this study, we shifted the focus to treatment effectiveness to assess its impact on the causal relationship between service quality and satisfaction. These results give insight as to how and which factors are involved in determination of medical service quality.

Although many studies have been conducted to evaluate satisfaction in conventional medicine hospitals, those pertaining to Korean medicine are rare [7]. As patient encounter time is much longer in Korean medicine hospitals compared to conventional medicine, it is highly likely that factors regarding treatment effectiveness and satisfaction are different. As most previous studies were limited and stopped at testing the hypotheses in the final model, we attempted to go one step further by examining path coefficients between exogenous latent variables affecting endogenous latent variables and analyzing indirect effects. We also used a large sample size that covered a wide area of geographic importance in Korea, overcoming the limitations of some of the previous studies conducted within a more limited range. This increases the study’s applicability to other Korean medicine hospitals. However, the greatest limitation of this study may be that the hypothesis and results of this study are still reflective of CAM practices in clinical settings within Korea. The fact that the survey was conducted at multiple sites of Jaseng Hospital of Korean medicine limits external validity in generalizing the results to other institutions or countries that use CAM. Countries outside of Korea may not have corresponding spine-specialty hospitals which employ the use of CAM, or healthcare systems that consider quality improvement in similar terms. Still, these findings will hopefully be of use to healthcare policy makers in countries or healthcare systems looking to improve the quality of CAM treatment. Consideration of expanding research to a wider range of medical institutions could be entertained to improve external validity of the model, and the study design should be developed further to secure sample representativeness and improve data accuracy [41]. As pathways may differ by hospital size, scope, and patient group (e.g. inpatient or ambulatory care), comparison of hospitals by structure and patient type could be of further interest [42]. Other limitations include potential interrater difference by survey site and selection bias in convenience sampling. Also, as results are highly subject to change by time in cross-sectional designs, more longitudinal studies are warranted.

Several studies have discussed the implications and methods for heightening application of survey results, and although patient feedback surveys are increasingly seen as a key factor in monitoring and improving the quality of medical services, assessments by patients based on physician-established criteria may not be a reliable basis for measuring the quality of patient care [43]. In addition, considering this incongruity, evaluation of medical service quality based only on patient feedback may not be sufficient foundation on which to instigate change. Still, analysis of customer satisfaction through service quality assessment has been purported to enable better prediction of customer behavior, contain customer reduction rates, and increase customer value [44, 45]. By increasing customer value, service quality improvements additionally increase customer satisfaction, cut financial costs [46, 47], and promote long-term customer relationships [48, 49]. Meanwhile, other studies have attested to how well-designed patient questionnaires can contribute to assessment of both the technical proficiency and interpersonal skills of doctors [50]. Specifics on which constructs scored lower in satisfaction rates are more informative for determining which service quality factors require improved performance as opposed to simply stating satisfaction was low. Therefore, to reach a full circle from survey to actual implementation towards quality improvement, clear factual results that prompt follow-up actions are needed.

The questionnaire used in this study was compiled for research means and to reflect hospital characteristics. Although analysis did not strictly follow the SERVQUAL format, most service quality items from SERVQUAL were included. Satisfaction was analyzed as a single item in the final model after excluding items impeding reliability and validity. Though items interfering with reliability do not necessarily have to be eliminated, they were ultimately excluded to further refine the model and establish higher quality evidence. Future studies should include a sufficient number of category items in preparation for possible exclusion in analyses, and consideration for application of results in service quality measurement and improvement should be given from the survey design stage.

## Conclusions

This nationwide survey study introduces the treatment effect-focused medical service quality evaluation results of a CAM-oriented spine-specialty Korean medicine hospital. Based on these results, we suggest the need for further research to assess whether this relationship plays different roles in Korean medicine and conventional medicine, and which physician-provided services are most significant in treatment effectiveness.

## Additional file

Additional file 1: Outpatient department customer satisfaction survey. The final questionnaire used for collection of data. (DOCX 252 kb)

## Abbreviations

AVE: Average variance extracted
CAM: Complementary and alternative medicine
CFA: Confirmatory factor analysis
IRB: Institutional Review Board
SE: Standard error
SEM: Structural equation modeling

## References

[1] Lewis RC, Booms B. The marketing aspects of service quality. In: *AMA proceedings.* Chicago: American Marketing Association; 1983. p. 99–104.

[2] Oliver RL, Balakrishnan PVS, Barry B (1994). Outcome satisfaction in negotiation: a test of expectancy disconfirmation. Organ Behav Hum Decis Process.

[3] Hall JA, Dornan MC (1988). Meta-analysis of satisfaction with medical care: description of research domain and analysis of overall satisfaction levels. Soc Sci Med.

[4] Lei P, Jolibert A (2012). A three-model comparison of the relationship between quality, satisfaction and loyalty: an empirical study of the Chinese healthcare system. BMC Health Serv Res.

[5] Chang C-S, Chen S-Y, Lan Y-T (2013). Service quality, trust, and patient satisfaction in interpersonal-based medical service encounters. BMC Health Serv Res.

[6] Barnes PM, Powell-Griner E, McFann K, Nahin RL. Complementary and alternative medicine use among adults: United States, 2002. Sem Integr Med. 2004;2(2):54–71. 15188733

[7] Seo Y-J, Kang S-H, Kim Y-H, Choi D-B, Shin H-K (2010). Systematic review on the Customers’ use of and satisfaction with oriental medical services. J Korean Oriental Med.

[8] W-k Y (2003). A study on recognition level of the people on oriental medical services and the need for its improvement. Korean J Oriental Prev Med Soc.

[9] Harris K, Baron S (2004). Consumer-to-consumer conversations in service settings. J Serv Res.

[10] Parasuraman A, Zeithaml VA, Berry LL. A conceptual model of service quality and its implications for future research. J Mark. 1985;49(4):41-50.

[11] Magill M, Mastroleo NR, Apodaca TR, Barnett NP, Colby SM, Monti PM (2010). Motivational interviewing with significant other participation: assessing therapeutic alliance and patient satisfaction and engagement. J Subst Abuse Treat.

[12] Vincent C (1990). Credibility assessment in trials of acupuncture. Complement Med Res.

[13] Brown WA (2015). How expectation works: psychologic and physiologic pathways. Rhode Island Med J (2013).

[14] Zeithaml VA, Berry LL, Parasuraman A. The behavioral consequences of service quality. J Mark. 1996;60(2):31-46.

[15] Kotler P, Armstrong G (2010). Principles of marketing.

[16] Reidenbach RE, Sandifer-Smallwood B (1990). Exploring perceptions of hospital operations by a modified SERVQUAL approach. Mark Health Serv.

[17] Woodside AG, Frey LL, Daly RT. Linking service quality, customer satisfaction, and behavioral intention. J Health Care Mark. 1989;9(4):5–17. 10304174

[18] Stevens L, Duarte H, Park J (2007). Promising implications for integrative medicine for back pain: a profile of a Korean hospital. J Altern Complement Med.

[19] Robinson N, Liu J (2012). Oriental and traditional medicine–supporting the vision for integrated health. Eur J Integr Med.

[20] Shin J-S, Lee J (2014). Kim M-r, Shin B-C, lee MS, ha I-H: **the long-term course of patients undergoing alternative and integrative therapy for lumbar disc herniation: 3-year results of a prospective observational study**. BMJ Open.

[21] Hair J, Anderson R (1995). Multivariate data analysis with reading.

[22] Babakus E, Mangold WG (1992). Adapting the SERVQUAL scale to hospital services: an empirical investigation. Health Serv Res.

[23] Cronin Jr JJ, Taylor SA. SERVPERF versus SERVQUAL: reconciling performance-based and perceptions-minus-expectations measurement of service quality. J Mark. 1994;58(1):125–31.

[24] Donabedian A (1980). The definition of quality and approaches to its Assessment. Vol 1. Explorations in quality Assessment and monitoring.

[25] Bagozzi RP, Yi Y (1988). On the evaluation of structural equation models. J Acad Market Sci.

[26] Kline RB (1998). Principles and practice of structural equation modeling.

[27] Nunnally J (1978). Psychometric theory.

[28] Zeithaml VA. Consumer perceptions of price, quality, and value: a means-end model and synthesis of evidence. J Mark. 1988;52(3):2–22.

[29] Grewal D, Monroe KB, Krishnan R. The effects of price-comparison advertising on buyers’ perceptions of acquisition value, transaction value, and behavioral intentions. J Mark. 1998;62(2):46–59.

[30] Beatty SE, Mayer M, Coleman JE, Reynolds KE, Lee J (1996). Customer-sales associate retail relationships. J Retail.

[31] Bendapudi N, Berry LL (1997). Customers’ motivations for maintaining relationships with service providers. J Retail.

[32] Abramowitz S, Coté AA, Berry E (1987). Analyzing patient satisfaction: a multianalytic approach. QRB Qual Rev Bull.

[33] Cleary PD, McNeil BJ. Patient satisfaction as an indicator of quality care. Inquiry. 1988;25(1):25–36. 2966123

[34] Doering ER (1983). Factors influencing inpatient satisfaction with care. QRB Qual Rev Bull.

[35] Park YU (1997). Factors affecting in-patient satisfaction of oriental hospital. J Korean Soc Health Educ.

[36] Cronin Jr JJ, Taylor SA. Measuring service quality: a reexamination and extension. J Mark. 1992;56(3):55–68.

[37] Butt MM, de Run EC (2010). Private healthcare quality: applying a SERVQUAL model. Int J Health Care Qual Assur.

[38] Lim PC, Tang NK (2000). A study of patients’ expectations and satisfaction in Singapore hospitals. Int J Health Care Qual Assur.

[39] Zarei A, Arab M, Froushani AR, Rashidian A, Tabatabaei SMG (2012). Service quality of private hospitals: the Iranian Patients’ perspective. BMC Health Serv Res.

[40] Richins ML, Bloch PH. After the new wears off: The temporal context of product involvement. J Consum Res. 1986;13(2):280–5.

[41] Kim JS (2013). The effects of elderly Patients’ dental satisfaction on revisit intention with the application of SEM (structural equation model).

[42] Kim SS, Jung CH (2012). The effects of service quality on service value, customer satisfaction, and revisit intention in healthcare services. Korean Res Assoc Bus Educ.

[43] Rao M, Clarke A, Sanderson C, Hammersley R (2006). Patients’ own assessments of quality of primary care compared with objective records based measures of technical quality of care: cross sectional study. BMJ.

[44] Reinartz WJ, Kumar V (2000). On the profitability of long-life customers in a noncontractual setting: an empirical investigation and implications for marketing. J Mark.

[45] Reinartz W, Kumar V (2002). The mismanagement of customer loyalty. Harv Bus Rev.

[46] Anderson JC, Narus JA. A model of distributor firm and manufacturer firm working partnerships. J Mark. 1990;54(1):42–58.

[47] Chowdhury S. The role of affect-and cognition-based trust in complex knowledge sharing. J Managerial Issues. 2005;17(3):310–26.

[48] Lovelock C, Wirtz J (2004). Services marketing: people, technology, strategy. J Serv Mark.

[49] Doney PM, Cannon JP. An examination of the nature of trust in buyer-seller relationships. J Mark. 1997;61(2):35–51.

[50] Coulter A (2006). Can patients assess the quality of health care?. BMJ.

