# Supplementary material for: Quality of medical service, patient satisfaction and loyalty with a focus on interpersonal-based medical service encounters and treatment effectiveness: a cross-sectional multicenter study of complementary and alternative medicine (CAM) hospitals
Source: BMC Complement Altern Med. 2017 Mar 28;17:174. doi: 10.1186/s12906-017-1691-6 (PMC5370429; doi:10.1186/s12906-017-1691-6)
Supplement: Additional file 1: — Outpatient department customer satisfaction survey. The final questionnaire used for collection of data. (DOCX 252 kb) [file 12906_2017_1691_MOESM1_ESM.docx]

**
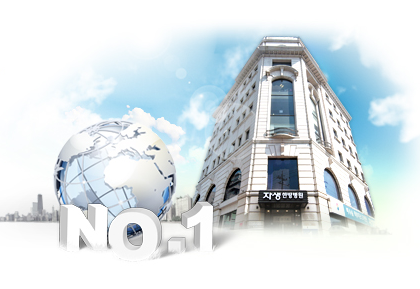
Customer satisfaction survey**

**We hope you find good health here at Jaseng Hospital of Korean medicine.**

**We thank you for your kind interest in and consideration of Jaseng Hospital of Korean medicine and promise to continuously strive to provide you with the best quality services and a comfortable and pleasant environment.**

**
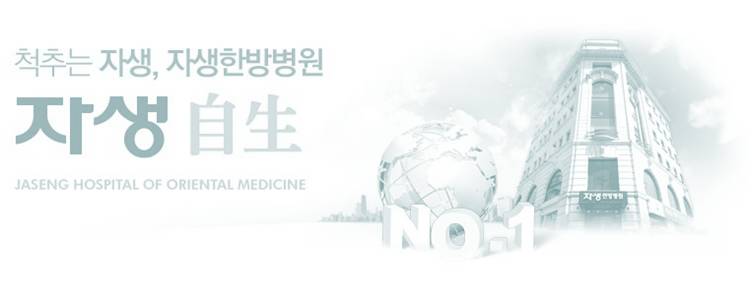
We are conducting a medical service satisfaction survey to refine the services we currently offer to patients and guardians. We cordially request that you kindly participate in the survey considering for the following.**

**Your answers will be collected and used only for statistical means, and individual item answers or identity will not be exposed. We would be highly grateful if you would kindly take the time to briefly answer this survey.**

**- Jaseng Hospital of Korean medicine -**

**
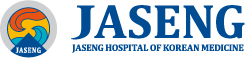
**

**Survey date: ______________________ Survey number: ________ Site code: ______**

*** Please select a score for the following questions.**

| **Ⅰ. These questions are on the facilities and environment of Jaseng Hospital of Korean medicine.**  **Please rate your level of satisfaction for each item.** | | **Ⅰ. Facilities and Environment satisfaction** | | | | |
| --- | --- | --- | --- | --- | --- | --- |
|  |  | **Very**  **satisfied** | **Satisfied** | **Neutral** | **Dissatisfied** | **Very**  **dissatisfied** |
| 1 | Hospital location and transportation was convenient. | 5 | 4 | 3 | 2 | 1 |
| 2 | The parking facility was convenient to use. | 5 | 4 | 3 | 2 | 1 |
| 3 | Hospital indoor temperature (air conditioning/heating) and ventilation was satisfactory. | 5 | 4 | 3 | 2 | 1 |
| 4 | The hospital was clean and pleasant overall. | 5 | 4 | 3 | 2 | 1 |
| 5 | The hospital was well-equipped with amenities (e.g. cafe, drink vending machine, water purifier, waiting space, cash machine). | 5 | 4 | 3 | 2 | 1 |
| 6 | On-site hospital facilities were easy to locate (e.g. consultation room, diagnostic imaging department, physical therapy room, restroom). | 5 | 4 | 3 | 2 | 1 |
| 7 | I am satisfied with the hospital facilities and environment in regards to all of the above. | 5 | 4 | 3 | 2 | 1 |

| 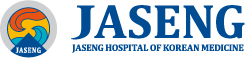 | Outpatient department customer satisfaction survey |
| --- | --- |

*** Please select a score for the following questions.**

| **Ⅱ. These questions are on the service procedures of Jaseng Hospital of Korean medicine.**  **Please rate your level of satisfaction for each item.** | | **Ⅱ. Service procedure satisfaction** | | | | |
| --- | --- | --- | --- | --- | --- | --- |
|  |  | **Very**  **satisfied** | **Satisfied** | **Neutral** | **Dissatisfied** | **Very**  **dissatisfied** |
| 1 | Making appointments was convenient. | 5 | 4 | 3 | 2 | 1 |
| 2 | I was able to make appointments on the date and time I wanted. | 5 | 4 | 3 | 2 | 1 |
| 3 | Staff was prompt in receiving and returning phone calls. | 5 | 4 | 3 | 2 | 1 |
| 4 | The registration procedure for consultations was convenient. | 5 | 4 | 3 | 2 | 1 |
| 5 | Adequate information on waiting time was given in advance. |  |  |  |  |  |
| 6-1 | Waiting time duration of examination and treatment were acceptable. | 5 | 4 | 3 | 2 | 1 |
| 6-2 | (To be answered upon selection of ‘dissatisfied’ or ‘very dissatisfied’ in 6-1)  How much waiting time would you say is acceptable? ㅁ( minutes) | | | | | |
| 7 | The payment process was convenient. | 5 | 4 | 3 | 2 | 1 |
| 8 | Payment receipt items were easy to understand. | 5 | 4 | 3 | 2 | 1 |
| 9 | I am satisfied with the hospital service procedures in regards to all of the above. | 5 | 4 | 3 | 2 | 1 |

| **Ⅲ. These questions are on the treatment (e.g. medicine, Chuna manual therapy, acupuncture) of Jaseng Hospital of Korean medicine.**  **Please rate your level of satisfaction for each item.** | | **Ⅲ. 치료 satisfaction** | | | | |
| --- | --- | --- | --- | --- | --- | --- |
|  |  | **Very**  **satisfied** | **Satisfied** | **Neutral** | **Dissatisfied** | **Very**  **dissatisfied** |
| 1 | Treatment was effective. | 5 | 4 | 3 | 2 | 1 |
| 2 | Treatment was reliable. | 5 | 4 | 3 | 2 | 1 |
| 3 | Treatment and prescriptions were appropriate. | 5 | 4 | 3 | 2 | 1 |
| 4 | Treatment cost was appropriate. | 5 | 4 | 3 | 2 | 1 |
| 5 | I am satisfied with hospital treatment in regards to all of the above. | 5 | 4 | 3 | 2 | 1 |

*** Please select a score for the following questions.**

| **Ⅳ. These questions are on the physicians, nursing staff, administrative personnel of Jaseng Hospital of Korean medicine.**  **Please rate your level of satisfaction for each item.** | | **Ⅳ. Physicians, nursing staff, administrative personnel satisfaction** | | | | |
| --- | --- | --- | --- | --- | --- | --- |
|  |  | **Very**  **satisfied** | **Satisfied** | **Neutral** | **Dissatisfied** | **Very**  **dissatisfied** |
| **<Physician satisfaction>** | | | | | | |
| 1 | The physicians were neat and tidy in appearance. | 5 | 4 | 3 | 2 | 1 |
| 2 | The physicians were kind and courteous. | 5 | 4 | 3 | 2 | 1 |
| 3 | Information on treatment was always given by physicians in advance. | 5 | 4 | 3 | 2 | 1 |
| 4 | The physicians were attentive to my conversation (queries). | 5 | 4 | 3 | 2 | 1 |
| 5 | The physicians gave sufficient explanation on symptoms and treatment plans that was easy to comprehend. | 5 | 4 | 3 | 2 | 1 |
| 6 | The physicians commanded sufficient professional knowledge. | 5 | 4 | 3 | 2 | 1 |
| 7 | I am satisfied with hospital physicians in regards to all of the above. | 5 | 4 | 3 | 2 | 1 |
| **<Nursing staff satisfaction>** | | | | | | |
| 1 | Nursing staff was neat and tidy in appearance. | 5 | 4 | 3 | 2 | 1 |
| 2 | Nursing staff was kind and courteous. | 5 | 4 | 3 | 2 | 1 |
| 3 | Nursing staff was attentive to my conversation (queries). | 5 | 4 | 3 | 2 | 1 |
| 4 | Nursing staff gave sufficient explanation on symptoms and treatment plans that were easy to comprehend. | 5 | 4 | 3 | 2 | 1 |
| 5 | Nursing staff commanded sufficient professional knowledge. | 5 | 4 | 3 | 2 | 1 |
| 6 | I am satisfied with hospital nursing staff in regards to all of the above. | 5 | 4 | 3 | 2 | 1 |
| **<Administrative personnel satisfaction>** | | | | | | |
| 1 | Administrative personnel were neat and tidy in appearance. | 5 | 4 | 3 | 2 | 1 |
| 2 | Administrative personnel were kind and courteous. | 5 | 4 | 3 | 2 | 1 |
| 3 | Administrative personnel were attentive to my conversation (queries). | 5 | 4 | 3 | 2 | 1 |
| 4 | Administrative personnel gave sufficient explanation on symptoms and treatment plans that were easy to comprehend. | 5 | 4 | 3 | 2 | 1 |
| 5 | My queries (demands) were promptly taken care of. | 5 | 4 | 3 | 2 | 1 |
| 6 | I am satisfied with hospital administrative personnel in regards to all of the above. | 5 | 4 | 3 | 2 | 1 |

**Ⅵ. Please select a score for the generalities of Jaseng Hospital of Korean medicine in view of all of the above.**

**1. Are you satisfied overall with Jaseng Hospital of Korean medicine?**

**① Very satisfied ㅁ② Satisfied ③ Neutral ④ Dissatisfied ⑤ Very dissatisfied**

**2. Are you satisfied with provided treatment services considering treatment costs?**

**① Very satisfied ㅁ② Satisfied ③ Neutral ④ Dissatisfied ⑤ Very dissatisfied**

**3. Do you intend to continue using Jaseng Hospital of Korean medicine?**

**① Definitely ㅁ② Probably ③ Neutral (Sometimes) ④ Rarely ⑤ Never**

**4-1. Do you intend to recommend Jaseng Hospital of Korean medicine to others? (Check corresponding score)**

**Definitely not Maybe Definitely**

**0 1 2 3 4 5 6 7 8 9 10**

**4-2. What is the reason for non-recommendation? (Multiple responses allowed)**

1. **Hospital service procedure was too complicated ② Treatment costs are too expensive**
2. **Employees are uncooperative ④ Hospital facilities are inferior**

**⑤ Treatment effects are undistinguishable ⑥ Waiting times are too long**

**⑦ Physicians are not highly skilled ⑧ Other ( )**

**Ⅶ. Lastly, please feel free to leave us any suggestions or comments on Jaseng Hospital of Korean medicine.**

|  |
| --- |

*** The following data is collected for statistical analysis and verification means.**

**1. Age: ① 20s ㅁ② 30s ③ 40s ④ 50s ⑤ 60s or older**

**2. Sex: ① Male ㅁ② Female**

**3. Residential area:** __________**City (Province)** __________**District (City)**

**4. Occupation: ① Student ㅁ② Housewife ③ Corporate worker ④ Civil servant ⑤ Entrepreneur ⑥ Other ( ) ⑦ No response**

**5. How many times have you received ambulatory treatment at Jaseng Hospital of Korean medicine?**

**① First visit ㅁ② 2^nd^ visit ③ 3^rd^ visit ④ 4^th^ visit ⑤ 5^th^ visit or more**

**Thank you for your participation**
